# The economic burden of malaria on households and the health system in a high transmission district of Mozambique

**DOI:** 10.1186/s12936-019-2995-4

**Published:** 2019-11-11

**Authors:** Sergi Alonso, Carlos J. Chaccour, Eldo Elobolobo, Amilcar Nacima, Baltazar Candrinho, Abuchahama Saifodine, Francisco Saute, Molly Robertson, Rose Zulliger

**Affiliations:** 1 Centro de Investigação em Saúde de Manhiça, Bairro Cambeve, Rua 12, Distrito da Manhiça, CP 1929 Maputo, Mozambique; 2 ISGlobal, Hospital Clínic, Universitat de Barcelona, Barcelona, Spain; 3 Centre for Primary Care and Public Health, Barts and The London School of Medicine & Dentistry, Queen Mary University of London, London, UK; 4 Programa Nacional de Controlo da Malaria (NMCP), Maputo, Mozambique; 5 U.S. President’s Malaria Initiative, US Agency for International Development, Maputo, Mozambique; 6 PATH, Washington, DC USA; 7 U.S. President’s Malaria Initiative and Malaria Branch, Division of Parasitic Diseases and Malaria, U.S. Centers for Disease Control and Prevention, Maputo, Mozambique

**Keywords:** Malaria, Mozambique, Household costs, Health system costs, Economic burden

## Abstract

**Background:**

Malaria remains a leading cause of morbidity and mortality in Mozambique. Increased investments in malaria control have reduced the burden, but few studies have estimated the costs of malaria in the country. This paper estimates the economic costs associated with malaria care to households and to the health system in the high burden district of Mopeia in central Mozambique.

**Methods:**

Malaria care-seeking and morbidity costs were routinely collected among 1373 households with at least one child enrolled in an active case detection (ACD) cohort in Mopeia, and through cross-sectional surveys with 824 families in 2017 and 805 families in 2018. Household costs included direct medical expenses, transportation and opportunity costs of the time lost due to illness. Structured questionnaires were used to estimate the health system costs associated with malaria care in all 13 district health facilities. Cost estimations followed an ingredient-based approach with a top-down allocation approach for health system expenses.

**Results:**

Among participants in cross-sectional studies, households sought care for nine severe malaria cases requiring hospital admission and for 679 uncomplicated malaria cases. Median household costs associated with uncomplicated malaria among individuals of all ages were US$ 3.46 (IQR US$ 0.07–22.41) and US$ 81.08 (IQR US$ 39.34–88.38) per severe case. Median household costs were lower among children under five (ACD cohort): US$ 1.63 (IQR US$ 0.00–7.79) per uncomplicated case and US$ 64.90 (IQR US$ 49.76–80.96) per severe case. Opportunity costs were the main source of household costs. Median health system costs associated with malaria among patients of all ages were US$ 4.34 (IQR US$ 4.32–4.35) per uncomplicated case and US$ 26.56 (IQR US$ 18.03–44.09) per severe case. Considering household and health system costs, the overall cost of malaria care to society was US$ 7.80 per uncomplicated case and US$ 107.64 per severe case, representing an economic malaria burden of US$ 332,286.24 (IQR US$ 186,355.84–1,091,212.90) per year only in Mopeia.

**Conclusions:**

Despite the provision of free malaria services, households in Mopeia incur significant direct and indirect costs associated with the disease. Furthermore, the high malaria cost on the Mozambican health system underscores the need to strengthen malaria prevention to reduce the high burden and improve productivity in the region.

## Background

Malaria remains a major global health challenge, causing an estimated 435,000 deaths globally in 2017, more than 60% of them children under five [1]. During the last decade, significant reductions in malaria morbidity and mortality have been achieved through increased funding for malaria control and elimination, among other reasons, which supported the scale-up of malaria prevention tools, such as insecticide-treated nets, and of case management, with rapid diagnostic tests (RDTs) and artemisinin-based combination therapy (ACT) [2].

Malaria is one of the main public health issues in Mozambique with an estimated 8.9 million cases and 14,700 deaths in 2017 [1], with children under five and pregnant women the most at risk groups. Zambézia province in central Mozambique is a high transmission area of malaria with an RDT-based prevalence of 68% in children under five in 2015 [3], and up to 47.8% in children below 15 years [4]. The peak transmission occurs during the rainy season (December to April), when the primary malaria vector species proliferate: *Anopheles gambiae* and *Anopheles funestus* [5]. Most of the cases are caused by *Plasmodium falciparum* infection [3], which may cause complications such as anemia, or cerebral malaria, particularly in children under five [6], in whom the majority of malaria deaths are concentrated [1].

In Mozambique, malaria is the leading cause of care-seeking, accounting for 45% of outpatient consultations and 24% of hospital admissions in 2015 [7]. The Mozambican Ministry of Health provides free diagnosis and treatment services for malaria at public health facilities [8], but these services require considerable human resource and health systems investment. Scarcity of resources and weak health system financing force the country to rely on international donors for the provision of RDTs and anti-malarial medicines, the majority of which are purchased by the US President’s Malaria Initiative and the Global Fund to Fight AIDS, Tuberculosis and Malaria.

Despite a policy of free universal access to malaria care in public facilities, patients still face several barriers, including limited access to malaria prevention and treatment [3], and sub-optimal malaria case management [9], because of few and distant health facilities with limited health services [10, 11]. Additionally, the poorest may suffer more from high indirect costs, such as transportation costs and long waiting times. They may also incur catastrophic payments when referred to the private or informal sector for treatment, due to recurrent stock-outs at public health facilities [11, 12].

The economic burden of malaria affects households, health systems and economic development and growth [13, 14]. Obtaining evidence on its economic burden has an important role in the economic evaluation of malaria control interventions and for the improved allocation of resources [15]. However, only a few studies have quantified the cost of malaria to families and the health system in Mozambique, and these have been conducted in the lower burden southern regions and in the context of evaluations of specific malaria interventions, such as intermittent prevention in infants or pregnant women [12, 16, 17]. No published studies are available on the cost of malaria in high burden areas in central or northern regions of Mozambique.

The objective of the present study is to determine the economic cost associated with malaria care on households, the health system, and society in the high malaria transmission district of Mopeia, Mozambique.

## Methods

This study was part of a larger project consisting of a cluster randomized-control trial (RCT) to determine the effectiveness and cost-effectiveness of implementing indoor residual spraying (IRS) with micro-encapsulated pirimiphos-methyl in an area with high coverage of long-lasting insecticidal bed nets (LLINs), as compared to LLIN coverage alone [5]. IRS was randomly assigned to 43 of 86 selected villages to receive free IRS with Actellic 300 CS (intervention arm) before the high malaria season in 2017 and 2018, while villages in the other clusters did not receive IRS (control arm). Randomization was stratified by cluster size and sampling utilized a ‘fried egg’ design [5]. Households in the intervention and control arms had been provided with free standard pyrethroid LLINs through mass distribution campaigns and continuous distribution. At baseline, 61–63% of households reported owning at least one LLIN and this increased to nearly all households following a universal coverage campaign of standard LLINs in 2017. Additional file 1 summarizes data sources used in the study and a cost categorization from which outcome variables are extracted.

### Study setting

The RCT was conducted in Mopeia district, one of 22 districts located in the Zambézia province of central Mozambique. At study baseline, Mopeia consisted of two administrative posts, eight localities, 104 villages and approximately 30,000 households [18]. The district projected population in 2014 was 151,570 individuals with 23,889 children under five years [18]. Mopeia has one health facility considered the district hospital and 12 clinics.

### Households’ costs estimation

Household costs of a clinical malaria case were determined through structured questionnaires among children during monthly visits in an active case detection (ACD) cohort and among individuals of all ages in cross-sectional studies. Sample sizes and power considerations for the ACD cohort, cross-sectional studies, and passive case detection (PCD) are reported elsewhere [5]. A total of 1536 children aged six months to five years old at baseline from 1373 households participated in an ACD cohort from January 2017 to October 2018. Trained fieldworkers conducted monthly ACD household visits that included malaria testing with an RDT and completion of structured questionnaires. They collected information on the household resources used for malaria care-seeking in order to determine the average household cost of an uncomplicated and severe malaria case for cohort children. Households were also asked how much time was lost to productive activities such as formal employment, farming, and childcare. Additionally, two cross-sectional studies undertook in May 2017 and April 2018 retrieved information on the resources used for malaria care-seeking among individuals of all ages using the same malaria cost questions as the ACD, but with reference to the entire household. Questionnaires were developed based on previous studies from the literature [19, 20].

Direct costs of malaria care-seeking from formal and informal services were collected and consisted of medical care—treatments, diagnostic tests, consultation fees—and non-medical care, such as travel and other costs (food and telephone expenses). While malaria care is provided for free in public facilities in Mozambique, households seek care at diverse settings. Costs included care from public health services (community health workers (CHWs) or health facilities), as well as informal care: traditional healers and self-treatment. Household costs are presented as aggregated costs under those categories as most of the households did not report costs related to consultation fees or malaria-related test and treatment costs. Indirect costs associated with malaria followed a wage-based valuation approach [21], consisting of computing the opportunity cost of the time loss for productive activities due to illness or caregiving. Such costs included missed work, agricultural activities, housework and school attendance. Productive time lost was measured in hours and multiplied by the corresponding Mozambican minimum wage according to the sector of employment [22, 23]. Severe malaria was defined as any case requiring hospital admission. The remaining malaria cases not requiring hospitalization were considered uncomplicated.

### Health system costs estimation

Provider costs associated with malaria care delivery were estimated through collecting information on clinical resources used during malaria outpatient visits and hospital admissions in the district health facilities. For uncomplicated malaria, a structured questionnaire was implemented in each of the 13 health facilities in July 2018. It retrieved information on the health facility characteristics, provision of health services, as well as resources used during a typical outpatient visit, including the health worker time devoted to patient care. The cost of screening suspected cases was also considered [20]: according to the RDT positivity rate of 50% obtained in the PCD; for each confirmed malaria case it was assumed another RDT was performed that was negative [1, 5]. For severe malaria, trained fieldworkers used a structured questionnaire to retrospectively collect data on the resources used for the management of malaria admissions from clinical files in the district hospital in 2016. These were the most recent data available at the time of data abstraction. Inclusion criteria for malaria admissions consisted of: (1) malaria as the primary admission cause, (2) evidence in the clinical file of a positive parasite-based diagnosis by RDT or microscopy, and (3) availability of the clinical file. Data were complemented with field interviews with health and administrative workers at the district level, to account for the resources used that were not documented in patient files, such as the allocation of overhead.

Economic costs to the health system followed an ingredient-based approach [24] in which all resources employed for the management of a malaria case were quantified, including all indirect costs related to those actions. Hence, for each input, outputs were computed as unit price (*p*) by the quantity employed (*q*). Economic costs consisted of the financial costs of resources paid by the Mozambican Ministry of Health and other resources provided by international donors, such as RDT or antimalarial medicines.

The average economic cost of an uncomplicated malaria case was computed as the mean cost of the resources used to manage a malaria outpatient in each of the district health facilities. The average economic cost of a severe malaria case was computed as the mean cost of admitted cases retrieved from the district hospital in 2016. For each admission, data on medical examinations, diagnostic tests and treatment dosages employed were collected from files. Admission was based on findings from laboratory and clinical examinations. Malaria diagnosis cost was computed as the unit price of the RDT and microscopy [25, 26] multiplied by the number of tests recorded on the patient file. In accordance with the national guidelines microscopy was assumed to be used to check for parasitological density each day during the admission. Malaria treatment was computed as the price per dose according to patient’s age, used as a proxy for weight [27–29]. A three-day course artemether–lumefantrine (AL) was assumed to follow parenteral therapy with artesunate [28]. The value of health worker’s time was added for both malaria diagnosis and treatment, as well as other non-malaria related tests. Diagnostic tests and treatments unitary costs were retrieved from official prices at the district hospital, including malaria commodities, whose unitary prices were similar to the value of those commodities purchased by international donors. Costs on salaries, overheads and other district health system expenditures were collected directly from the hospital administration. Prices not available from Mopeia’s District Directorate of Health, such as blood transfusion costs, were obtained by a literature search or international prices databases [30–32]. A 100% freight mark-up was applied to all commodities.

Health system costs included the allocation of Mopeia health system expenditures following a top-down allocation approach in which annual expenses in 2016 were distributed according to the proportion of the total number of malaria outpatients and admissions in relation to all-cause outpatients and admissions [33]. District overhead resources consisted of the cost of administrative personnel salaries and benefits, office supplies, district official transportation and per diems, utilities and other goods. Utility costs included energy and water supply, waste management, cleaning or security costs at health facilities and the district office. According to Mopeia’s District Directorate of Health (personal communication), the inflation-adjusted annual budget attributable to overheads was US$ 175,354 in 2016. The amount was imputed to a cost per uncomplicated malaria case according to the proportion of malaria cases registered in the district in 2016 (39,758) among all outpatient consultations (170,121). For severe malaria, the inpatient utilization rate of malaria over the total number of cases (206/780) was applied to the district annual budget. That amount was further divided by the number of malaria admissions (206) to account for the overhead costs associated to a malaria admission. Finally, it was divided by 365 to obtain the daily hospitalization cost per malaria admission (US$ 0.62), which was later multiplied by the number of admission days for each patient to obtain the median hospitalization cost per malaria admission [34].

### Economic burden of malaria

The value of resources associated with malaria was assessed following a societal perspective, which included the resources used by the healthcare provider and households, as well as the opportunity cost due to illness or caregiving [35, 36]. Thus, the societal mean and median cost associated with malaria was computed as the sum of household costs per case plus health system costs per case. The societal cost of uncomplicated and severe malaria was calculated separately for children under five and for all ages. The total economic burden of uncomplicated and severe malaria among households and the health system in the district was further estimated by multiplying the registered number of malaria cases in Mopeia in 2016 by the median societal costs.

### Statistical analysis

Stata v15 (Statacorp) was used for data analysis. The time horizon for the average cost associated with uncomplicated and severe malaria was the complete episode duration for which care was provided under the household and health system perspectives. Costs were initially retrieved in Mozambican meticais, inflation adjusted and later converted to 2018 US dollars (US$). The exchange rates used were 63.03 metical/US$ in 2016 and 63.53 metical/US$ in 2017 [37]. Costs were adjusted by applying the inflation rates of 12.3% in 2016 and 9.8% in 2017 [38].

The median and the interquartile-range (IQR), in addition to the mean values in tables, are reported for households and health system costs to provide a deeper understanding of cost estimates, due to the typical skewed distribution of cost data. Mann–Whitney-Wilcoxon test was used to identify statistically significant differences in the mean health system cost between: (1) gender; (2) children under and above five years of age admitted due to malaria; and (3) admissions depending on malaria season: rainy season being from October to June [4]. Sample power of cited non-parametric tests were calculated using bootstrap simulation with 1000 replications [15, 39].

Uncertainty was handled carrying out a univariate sensitivity analysis to assess the robustness of results by changing values of key parameters: increasing/decreasing 50% the (1) price of malaria treatment (AL and artesunate), (2) price of RDT and (3) overhead expenses; increasing/decreasing 30% the (1) Mozambican minimum wage and (2) health worker wage at the health facilities; as well as considering no cost of screening fever.

## Results

### Health system characteristics

As shown in Additional file 2, most of the 13 health facilities in the district had catchment areas of 5001–10,000 inhabitants. Only the district hospital had more than ten beds, including maternity services, but three health facilities had laboratories to collect and process samples. One car and one motorbike were fully operational in the district hospital, while a car was also available in one other facility. The remaining facilities had access to these district vehicles or others lent by NGOs or governmental institutions.

Health providers in Mozambique include medical doctors, medical technicians, medical assistants and nurses; as well as pharmacists and laboratory technicians. However, human resources in the district health facilities were scarce. Only one medical doctor was present in Mopeia. There were also six medical technicians, each one in a different health facility. Preventive medicine technicians (9/13) and medical assistants (7/13) were present in most of facilities, but few pharmacists (4/13) and laboratory technicians (3/13) worked in the facilities. Nurses were available in all health facilities. A total of 22 CHWs complemented the work of health professionals at the district facilities. Their malaria-related activities consisted mainly of testing and treating patients at the community level.

Regarding malaria commodities, all health facilities had RDTs in stock at the moment of the interview but three out of 13 health facilities reported that stock-outs occurred during the last three months. However, at the time of data collection AL stock-outs were common for different formulation packages: AL 20 mg/120 mg (total course of six tablets) and AL 20 mg/120 mg (18 tablets) were out-of-stock in three health facilities and AL 20 mg/120 mg (12 tablets) and AL 20 mg/120 mg (24 tablets) in two. At least one formulation of AL was out-of-stock during the last three months in ten facilities (Additional file 2). Common practice in Mozambique is to cut or combine packaging at the facility level when a formulation is stocked out to provide patients with the correct dosage.

### Household costs associated with malaria

Table 1 shows the costs of malaria care-seeking from the cross-sectional study population, which included all ages. A total of 818 households in 2017 and 801 in 2018 reported nine severe malaria cases and 679 uncomplicated malaria cases, all non-fatal, of which 514 (76%) sought treatment at a health facility or another source of care, such as traditional healer or CHW. Few households reported non-zero costs at the traditional healer (n = 11: median = US$ 2.52, IQR = US$ 1.73–6.04), or at the CHW (n = 54: median = US$ 0.07, IQR = US$ 0.03–0.16). Most of the households reported no costs (469 out of 679) or less than US$ 1 (675 out of 679) concerning treatment costs purchased at private or public providers. When aggregating all cost sources, the median household cost was US$ 3.46 (IQR US$ 0.07–22.41) per uncomplicated malaria case, while the median household cost was US$ 81.08 (IQR US$ 39.34–88.38) per severe malaria case. The mean costs were considerably higher due to some outliers who had much higher indirect costs. Indirect costs were the most important source of costs among households, representing most of the cost burden.

**Table 1:** Household cost per malaria case among individuals of all ages (2018 US$)

	Uncomplicated malaria care-seeking costs (n = 679) including outpatient consultations (n = 514)				Severe malaria care-seeking costs (n = 9)			
	Mean	SD	Median	IQR	Mean	SD	Median	IQR
Traditional healer (including transportation)	0.29	5.29	0.00	0.00–0.00	0.00	0.00	0.00	0.00–0.00
CHW costs	0.01	0.09	0.00	0.00–0.00	0.00	0.00	0.00	0.00–0.00
Travel costs to/from the health facility	0.34	1.21	0.00	0.00–0.00	1.16	2.23	0.00	0.00–0.69
Direct costs at the health facility	0.05	0.10	0.03	0.00–0.06	2.16	2.22	2.19	0.09–3.57
Treatment costs after health facility visit	0.04	0.15	0.00	0.00–0.03	0.23	0.36	0.06	0.00–0.16
*Subtotal: direct costs of care-seeking*	*0.74*	*5.47*	*0.05*	*0.00–0.19*	*3.54*	*2.93*	*3.75*	*0.60–6.31*
Value of time lost in the main economic activity	16.67	31.17	2.29	0.00–22.38	154.30	192.89	76.97	31.45–87.71
Total costs	17.41	31.69	3.46	0.07–22.41	157.85	194.43	81.08	39.34–88.38

Data from cross-sectional studies

The value of the direct costs of care-seeking is calculated as the summation of the costs related to the traditional healer, CHW, travel to/from the health facility and treatments after a health facility visit

*CHW* community health worker, *IQR* interquartile-range, *SD* standard deviation

Additionally, a cohort of 1536 children in 1373 households was followed monthly from January 2017 to October 2018. As shown in Table 2, caregivers reported care-seeking costs associated with malaria in a child under five for a total of 2519 malaria cases, of which 1224 (49%) sought care at a health facility. This lower care-seeking behavior was likely influenced by the treatment received by study participants during ACD visits. The median household cost was US$ 1.63 (IQR US$ 0.00–7.79) per uncomplicated malaria case. These costs were mainly due to indirect costs (US$ 0.33) rather than direct cost of care-seeking (US$ 0.01). Additionally, ten households reported costs associated with severe malaria at the district hospital, which required hospitalization. The median household cost was US$ 64.90 (IQR US$ 49.76–80.96) per severe malaria case. These costs were greatly explained by indirect costs (US$ 59.37) as median direct costs of care-seeking were US$ 2.90 per severe malaria case. For both, ACD and cross-sectional cohort, agriculture and housework were by far the main sectors of activity. Average median costs were sensitive to variations in minimum wages; in particular an increase of minimum wages in 30% would increase the household cost per uncomplicated malaria case by 18% and per severe malaria case by 10% (Additional file 3).

**Table 2:** Household cost per malaria case among children under the five (2018 US$)

	Uncomplicated malaria care-seeking costs (n = 2519) including outpatient consultations (n = 1224)				Severe malaria care-seeking costs (n = 10)			
	Mean	SD	Median	IQR	Mean	SD	Median	IQR
Traditional healer (including transportation)	0.14	1.53	0.00	0.00–0.00	0.00	0.00	0.00	0.00–0.00
CHW costs	0.01	4.27	0.00	0.00–0.00	0.00	0.00	0.00	0.00–0.00
Travel costs to/from the health facility	0.72	1.32	0.00	0.00–0.94	0.95	1.24	0.41	0.00–1.38
Direct costs at the health facility	0.03	0.16	0.00	0.00–0.02	2.98	2.53	1.85	1.21–5.50
Treatment costs after health facility visit	0.02	0.11	0.00	0.00–0.00	0.75	2.22	0.08	0.00–0.09
*Subtotal: direct costs of care-seeking*	*0.92*	*4.48*	*0.01*	*0.00–1.05*	*4.68*	*4.36*	*2.90*	*1.99–6.31*
Value of time lost in main economic activity for the primary caregiver	7.23	*17.17*	0.33	0.00–5.90	61.23	*31.45*	59.37	47.17–71.60
Total costs	8.15	18.22	1.63	0.00–7.79	65.91	32.05	64.90	49.76–80.96

Data from active case detection (ACD) surveillance system

The value of the direct costs of care-seeking is calculated as the summation of the costs related to the traditional healer, CHW, travel to/from the health facility and treatments after a health facility visit

*CHW* community health worker, *IQR* interquartile-range, *SD* standard deviation

### Health system costs associated with malaria

Clinical practices for the management of uncomplicated malaria were similar in all district health facilities and severe cases were transferred to the district hospital for admission. Outpatient consultations for uncomplicated malaria in all 13 health facilities were diagnosed through RDTs and prescribed AL, in accordance with national guidelines [1, 40]. The average health system cost was US$ 4.34 (IQR US$ 4.32–4.35) per uncomplicated malaria case, mostly due to costs related to malaria diagnosis (39%), which incorporated the extra cost of screening fever, and treatment (36%) (Table 3). Allocation of overhead reached a median cost of US$ 1.03 per uncomplicated malaria case [34].

**Table 3:** Health system costs per uncomplicated malaria case among patients of all ages (2018 US$)

Cost category	Mean	SD	% of total (%)	Median	IQR
Medical examinations	0.06	0.52	1.38	0.05	0.03–0.10
Malaria diagnostic tests	1.67	0.09	38.48	1.66	1.60–1.74
Malaria treatment	1.57	0.69	36.18	1.54	1.51–1.64
Overhead cost allocation	1.03	–	23.18	1.03	–
Total costs	4.34	0.09		4.34	4.32–4.35

N = 13

*IQR* interquartile-range, *SD* standard deviation

Data from 107 malaria admissions were retrieved for the year 2016; almost half of them were children under five (52) and females (51) (Additional file 4). Malaria diagnosis and monitoring included microscopy in 72% of cases and RDTs in 67% of them. The vast majority of patients were treated with artesunate (84%) and, to a lesser extent, quinine (15%). Patients required a median of five days of hospitalization, most cases were discharged successfully (81%) but 10% died during hospitalization. As shown in Table 4, the median cost per severe malaria case was US$ 26.56 (mean = US$ 36.97). Medical examinations, diagnostics and treatments and provider costs increased the cost due to the days spent hospitalized. The daily hospitalization cost was US$ 0.62, which lead to a median hospitalization cost of US$ 3.80 (IQR US$ 2.28–12.15) per malaria admission. As shown in Additional file 3, univariate price variations in malaria commodities or overhead expenditures would increase up to 18% the health system cost per uncomplicated malaria case, but would have limited impact on the cost per severe malaria case. No differences in severe malaria costs were found between sex groups, but costs due to severe malaria were statistically significantly higher in children under five and in patients admitted during the dry season (Additional file 5).

**Table 4:** Health system costs per severe malaria case among patients of all ages (2018 US$)

Cost category	Mean	SD	Median	IQR
Medical examinations	3.44	0.59	3.28	3.09–3.57
Health facility transfer	4.24	22.44	0.00	0.00–0.00
Malaria diagnostic tests	8.51	3.94	7.85	5.40–12.17
Non-malaria related tests	0.52	5.27	0.00	0.00–0.00
Malaria treatment	5.00	9.41	2.42	1.56–6.32
Non-malaria related treatments	4.50	3.81	3.60	1.15–7.17
Hospitalization costs due to malaria admission^a^	10.75	25.84	3.80	2.28–12.15
Total costs	36.97	38.87	26.56	18.03–44.09

N = 107

*IQR* interquartile-range, *SD* standard deviation

^a^Hospitalization costs were computed as the overhead costs allocated to an admission day due to malaria multiplied by the number of hospitalization days

### Economic burden of malaria

As shown in Table 5, mean societal costs of malaria were US$ 21.75 per uncomplicated case and US$ 194.82 per severe case among patients of all ages. Costs were lower for children under five. Considering these costs and the registered number of malaria case in Mopeia in 2016 (39,758 outpatients and 206 admissions), the total economic burden of malaria in the district reached a median of US$ 332,286.24 (IQR US$ 186,355.84–US$ 1,091,212.90), mainly due to uncomplicated (median of US$ 310,112.40, IQR US$ 174,537.62–US$ 1,063,924.08) rather than severe malaria (median of US$ 22,173.84, IQR US$ 11,818.22–US$ 27,288.82).

**Table 5:** Mean, median and interquartile rage (IQR) societal costs associated with a malaria case by age and disease severity (2018 US$)

Severity	Mean	Median	IQR
Individuals of all ages			
Uncomplicated malaria	21.75	7.80	4.39–26.76
Severe malaria	194.82	107.64	57.37–132.47
Children under five			
Uncomplicated malaria	12.49	5.97	4.32–12.14
Severe malaria	102.88	91.46	67.79–125.05

## Discussion

Understanding the household, health system and societal economic burden of malaria is essential to informing policy decisions and evaluating new interventions to support malaria control and elimination. This kind of information is particularly relevant in regions like Mopeia, where malaria transmission is higher and poorer individuals are the most at risk to the disease [3, 41].

This study estimates the economic burden of malaria to households, the health system and society in a high transmission setting in which malaria services are provided free of charge at public health facilities. Given the rural and poor context of Mopeia [18], household costs associated with malaria care may constitute a significant drain on limited financial resources among the population as the incidence of poverty reached 60% of Zambézia population in 2014 [42]. A 2015 study reported a mean monthly expenditure per capita of 809 meticais in the province of Zambézia (approximately US$ 16.85) [43]. Hence, the median household cost associated with uncomplicated malaria represents between 10% (US$ 1.63) and 21% (US$ 3.46) of the monthly expenditure of a family in Zambézia, while the cost of a severe case (US$ 64.90 and US$ 81.08) exceeds the mean monthly expenditure per capita by more than three times. These amounts might be considered catastrophic expenditures, considering the threshold of 10% of total expenditure on healthcare, especially if more than one family member is infected with malaria [44]. As such, malaria is likely a driver of a vicious cycle of poverty in this high burden setting [45].

The median health system cost associated with malaria in Mopeia district was US$ 4.34/uncomplicated and US$ 26.56/severe malaria case. Despite obtaining slightly higher uncomplicated malaria costs, estimations are in line with those reported in a systematic review by White et al. in 2011 [46]; as well as those reported in Tanzania, Kenya and Ghana; ranging from US$ 1.75–2.89/uncomplicated malaria case [19]. However, estimations were higher in Nigeria (US$ 30.42–31.49/uncomplicated and US$ 48.02/severe case) [47, 48], Burkina Faso (US$ 6.74/uncomplicated and US$ 74.29/severe case) [49], and Kenya (US$ 95.58/severe case) [50]. These discrepancies may be driven by country-specific characteristics of the health system, such as higher budget for hospital activities, laboratory and pediatric wards funding [48, 49], or the quality of care or number of tests received during the admission [20, 48], underscoring the relevance of having local data to guide cost-effectiveness analyses.

This study described the contribution of each type of cost to the overall economic burden of malaria. Among direct costs, medical costs were higher during admissions due to the additional medical interventions received. Medical costs increased with age due to the higher dosage required. This translated into higher costs for the use of antimalarial medicines among adults, which has previously been reported [51]. As expected, households’ indirect costs were higher in the cross-sectional studies (all ages) compared to the ACD cohort (children under five) due to the fact that older individuals reported higher opportunity costs. Household care-seeking costs obtained in Mopeia are similar to the ones presented from other studies in sub-Saharan Africa. Sicuri et al. estimated the average cost of uncomplicated malaria ranging from US$ 3.56/case in Tanzania to US$ 8.68/case in Kenya, while for severe malaria ranged lower from US$ 19.82/hospitalization in Tanzania to US$ 48.73/hospitalization in Ghana [19]. Indirect costs were also the main source of costs to households. Similar findings were obtained in Nigeria (US$ 12.57/outpatient and US$ 23.20/admission) [47] and Malawi (US$ 17.48/admission) [52].

All health facilities in Mopeia followed similar practices for the management of uncomplicated malaria. Thus, most of the variability obtained for the estimation of an uncomplicated malaria case was due to differences in salaries of the health worker examining the patient. Conversely, the cost associated with severe malaria highly depended on the disease severity, which affected the length of stay and number of examinations undertaken by the health worker (both representing almost half of the total cost). Moreover, inter-hospital transfers, particularly those transferred to the provincial capital of Quelimane, represented up to 10% of the mean cost per severe malaria case even though only 8% of cases were transferred. Inter-hospital transfers, albeit expensive, remain cheaper than expanding the management of severe malaria to lower level hospitals, but transfer health risks and costs to patients [53, 54].

The mean economic burden of malaria to society reached US$ 21.75/uncomplicated and US$ 194.82/severe malaria case in patients of all ages, respectively, representing approximately 5% and 47% of the Mozambican average annual income of US$ 415 per capita in 2017 [55]. However, Zambézia is among the poorest regions in the country, with some estimates pointing to an average annual income of US$ 254 in 2012 [56]. Thus, these costs provide evidence on the high economic burden of malaria to households and the health system in high transmission regions, such as Mopeia, and highlight the importance of malaria prevention to reduce the high morbidity and mortality rates and improve overall productivity of the region [57].

Several limitations are associated with this study. First, self-reported household cost data were retrieved through cross-sectional and ACD studies and not through exit surveys at health facilities. Thus, recall bias and the context of the study may have influenced the responses regarding the actual resources spent during a malaria case [58]. Second, the assumption that AL was prescribed for all patients attending a health facility might involve double-counting treatment costs. However, double-counting bias has a limited impact over total costs: 70% of cases in the cross-sectional cohort and 80% of them in the ACD cohort reported no treatment costs, and most of those reporting costs were lower than US$ 1. On the contrary, the economic cost associated with malaria is likely to be underestimated as certain costs were disregarded: costs of mortality and of other disease manifestations, such as anemia, or other long-term sequelae such as residual disability from severe malaria. Similarly, household care-seeking costs might be higher than the ones obtained here: study participants received anti-malarial treatment when they had a positive test result, reducing their need for care-seeking: only 49% of malaria cases sought care at a health facility in the ACD cohort. Additionally, few households reported costs at the traditional healer (0.75% and 1.62% of ACD and cross-sectional cohort, respectively) or CHW (5% and 8% of ACD and cross-sectional cohort, respectively), thus limiting a disaggregated analysis by healthcare provider. Finally, these results cannot be generalized to other settings or countries. However, generalization was not a target of the present study, whose main purpose was to quantify the economic burden of malaria for the economic evaluation of malaria prevention intervention(s) carried out in the district.

## Conclusions

This study provides evidence of the large economic cost of malaria care in a high transmission region of Mozambique, where data on the societal cost of malaria are scarce. The results demonstrate the substantial economic burden imposed by malaria among the households in Mopeia who already confront financial vulnerability. This study also shows the important financial constraint that malaria represents for the Mozambican health system, particularly in high transmission and difficult to reach regions. These findings provide important parameter values for the determination of cost-effectiveness of malaria prevention and control interventions such as IRS. More importantly, the results underscore the importance of investing in malaria prevention to reduce the high malaria burden and improve productivity.

## Supplementary information

**Additional file 1.** Data sources and cost categorization.

**Additional file 2.** Description of 13 health facilities of Mopeia district.

**Additional file 3.** Results of the univariate sensitivity analysis.

**Additional file 4.** Management of severe malaria at district hospital.

**Additional file 5.** Differences in health system costs due to severe malaria according to sex, age and malaria season.

## Data Availability

The datasets used and/or analyzed during the current study are available from the corresponding author on reasonable request.

## Abbreviations

ACD: active case detection
ACT: artemisinin-based combination therapy
AL: artemether–lumefantrine
CHW: community health worker
IQR: interquartile range
IRS: indoor residual spraying
LLIN: long-lasting insecticidal bed net
PCD: passive case detection
RCT: randomized-control trial
RDT: rapid diagnostic test
US$: United States Dollar
WHO: World Health Organization
