# Supplementary material for: The economic burden of malaria on households and the health system in a high transmission district of Mozambique
Source: Malar J. 2019 Nov 11;18:360. doi: 10.1186/s12936-019-2995-4 (PMC6849240; doi:10.1186/s12936-019-2995-4)
Supplement: Supplementary file 1 — Additional file 1. Data sources and cost categorization. [file 12936_2019_2995_MOESM1_ESM.docx]

| **Additional file 1.** Data sources and cost categorization. ACD: active case detection, AL: artemether-lumefantrine, CHW: community health worker, HF: health facility, RDT: rapid diagnostic test. | | | | | | |
| --- | --- | --- | --- | --- | --- | --- |
| **Data source** | **Observations** | **Years** | **Target** | **Elicitation** | **Outcomes** | **Cost categories and other information** |
| **Household costs associated with malaria** | | | | | | |
| ACD follow-up | 688 cases | 2016-2018 | Children 6 months to 5 years | Self-reported | Uncomplicated and severe malaria case | Traditional healer^1^, CWH^1^, transport to HF, direct medical^2^ and non-medical^3^ at HF, treatment after the HF and indirect costs. |
| Cross-sectional | 2,529 cases | 2017-2018 | All ages |  |  |  |
| **Health system costs associated with malaria** | | | | | | |
| Retrospective patient data | 107 admissions | 2016 | Malaria admissions | Clinical record | Severe malaria case | Medical interventions registered in files: treatment^4^ and diagnostic tests (including non-malaria related), time of health worker, and medical commodities employed.  Symptoms, number of days and outcome of the admission.  Socio-demographic data from patient: sex, age, weight and pregnancy status. |
| HF questionnaire | 13 key informants (one per HF) | 2018 | Malaria outpatients | Interview | Uncomplicated malaria case and health facility description | HF catchment area, human resources and services provided, access to vehicles, RDT and AL availability, health workers time to malaria activities and commodity utilization. |
| Notes:  ^1^Costs included user fees, diagnostics, treatment prescribed and transport to care.  ^2^Costs included user fees, diagnostics, treatments and information about admission expenses.  ^3^Costs included food, phone calls and others.  ^4^Treatments included dosage and intake frequency. | | | | | | |
