# Supplementary material for: The economic burden of malaria on households and the health system in a high transmission district of Mozambique
Source: Malar J. 2019 Nov 11;18:360. doi: 10.1186/s12936-019-2995-4 (PMC6849240; doi:10.1186/s12936-019-2995-4)
Supplement: Supplementary file 2 — Additional file 2. Description of 13 health facilities of Mopeia district. [file 12936_2019_2995_MOESM2_ESM.docx]

| **Additional file 2**. Description of 13 health facilities of Mopeia district. HF: health facility; RDT: rapid diagnostic test |
| --- |
| \|  \| \|  \| Freq \| % \|  \|  \| Freq \| % \| \| --- \| --- \| --- \| --- \| --- \| --- \| --- \| --- \| --- \| \| ***Facilities*** \|  \| \|  \|  \| ***Human resources*** \|  \|  \|  \| \| Health Facility (HF) catchment area (population) \| 0-5,000 \| \| 1 \| 8% \| # HF with medical doctor (general practitioner): *6 years of training (higher education)* \| \| 1 \| 8% \| \|  \| 5,001-10,000 \| \| 7 \| 54% \| # HF with medical doctor (preventive medicine): 6 *years of training (higher education)* \| \| 0 \| 0% \| \|  \| 10,001-15,000 \| \| 2 \| 15% \| # HF with medical technician (general medicine): *2.5 years of training (secondary education)* \| \| 6 \| 46% \| \|  \| 15,001-50,000 \| \| 0 \| 0% \| # HF with medical technician (preventive medicine): *2.5 years of training (secondary education)* \| \| 9 \| 69% \| \|  \| >50,000 \| \| 3 \| 23% \| # HF with nurse: *2.5 years of training (secondary education)* \| \| 13 \| 100% \| \| Working hours \| 7:00 to 15:00 \| \| 12 \| 92% \| # HF with pharmacist \| \| 4 \| 31% \| \|  \| 7:00 to 21:00 \| \| 1 \| 8% \| # HF with laboratory technician \| \| 3 \| 23% \| \| # HF with more than 10 beds \|  \| \| 1 \| 8% \| # HF with medical assistant: *1.5 years of training (secondary education)* \| \| 7 \| 54% \| \| # HF with laboratory \|  \| \| 3 \| 31% \| # HF with other worker: *(primary education)* \| \| 9 \| 69% \| \| # HF with cars \|  \| \| 2 \| 15% \|  \|  \|  \|  \| \| # HF with motorbikes \|  \| \| 1 \| 8% \|  \|  \|  \|  \| \| ***Commodities*** \|  \| \|  \|  \|  \|  \|  \|  \| \| # HF with stock of RDT at time of survey \|  \| \| 13 \| 100% \| # HF with stockout of AL 2x6 any day during last 3 months \| \| 9 \| 69% \| \| # HF with stockout of RDT any day during last 3 months \| \| \| 3 \| 23% \| # HF with stock of AL 3x6 at time of survey \| \| 10 \| 77% \| \| # HF with stock of AL 1x6 at time of survey \| \| \| 10 \| 77% \| # HF with stockout of AL 3x6 any day during last 3 months \| \| 9 \| 69% \| \| # HF with stockout of AL 1x6 any day during last 3 months \| \| \| 10 \| 77% \| # HF with stock of AL4x6 at time of survey \| \| 11 \| 85% \| \| # HF with stock of AL 2x6 at time of survey \| \| \| 11 \| 85% \| # HF with stockout of AL 4x6 any day during last 3 months \| \| 5 \| 38% \| |
