# Supplementary material for: The economic burden of malaria on households and the health system in a high transmission district of Mozambique
Source: Malar J. 2019 Nov 11;18:360. doi: 10.1186/s12936-019-2995-4 (PMC6849240; doi:10.1186/s12936-019-2995-4)
Supplement: Supplementary file 3 — Additional file 3. Results of the univariate sensitivity analysis. [file 12936_2019_2995_MOESM3_ESM.docx]

| **Additional file 3.** Results of the univariate sensitivity analysis: impact of changes on selected parameters on median outcome variables: median household cost per (1) uncomplicated and (2) severe malaria case; median health system cost per (3) uncomplicated and (4) severe malaria case. Absolute value in US$ (percentage change). ACT: artemisinin-based combination therapy; AL: artemether-lumefantrine; AS: artesunate; RDT: rapid diagnostic test | | | | |
| --- | --- | --- | --- | --- |
| Parameter | Median household cost (all ages) | | Median health system cost | |
|  | Uncomplicated malaria (US$3.46) | Severe malaria  (US$81.08) | Uncomplicated malaria (US$4.34) | Severe malaria  (US$26.56) |
| ACT (AL and AS) costs 50% higher | Negligible | Negligible | 5.08  (+17%) | 28.34  (+7%) |
| ACT (AL and AS) costs 50% lower | Negligible | Negligible | 3.59  (-17%) | 23.85  (-10%) |
| RDT 50% higher | Negligible | Negligible | 5.12  (+18%) | 27.23  (+3%) |
| RDT 50% lower | Negligible | Negligible | 3.54  (-18%) | 25.87  (-3%) |
| Mozambican minimum wages 30% higher | 4.09  (+18%) | 89.13  (+10%) | -- | -- |
| Mozambican minimum wages 30% lower | 2.52  (-27%) | 63.72  (-21%) | -- | -- |
| Health worker wages 30% higher | -- | -- | 4.36  (+0.5%) | 28.56  (+8%) |
| Health worker wages 30% lower | -- | -- | 4.30  (-1%) | 24.39  (-8%) |
| Overhead costs 50% higher | -- | -- | 4.85  (+12%) | 28.97  (+9%) |
| Overhead costs 50% lower | -- | -- | 3.82  (-12%) | 24.45  (-8%) |
| No cost of screening fever | -- | -- | 3.54  (-18%) | -- |
