# Supplementary material for: The economic burden of malaria on households and the health system in a high transmission district of Mozambique
Source: Malar J. 2019 Nov 11;18:360. doi: 10.1186/s12936-019-2995-4 (PMC6849240; doi:10.1186/s12936-019-2995-4)
Supplement: Supplementary file 4 — Additional file 4. Management of severe malaria at district hospital. [file 12936_2019_2995_MOESM4_ESM.docx]

| **Additional file 4.** Management of severe malaria at district hospital **(N=107)**.  AL: artemether-lumefantrine; AS: artesunate; IQR: interquartile-range |
| --- |
| \| **Malaria admissions (N=107)** \|  \| Freq \| % \| \| --- \| --- \| --- \| --- \| \| **Age range** \| <5 years \| 52 \| 49% \| \|  \| 5-15 years \| 9 \| 8% \| \|  \| >15 years \| 46 \| 43% \| \| **Sex** \| Female \| 51 \| 48% \| \| **Transferred from peripheral health facility** \| Yes \| 9 \| 8% \| \| **Blood transfusion** \| Yes \| 1 \| 1% \| \| **Rapid diagnostic test (RDT)** \| Yes \| 67 \| 63% \| \| **Microscopy** \| Yes \| 77 \| 72% \| \| **Antimalarial therapy** \| Artesunate (AS) + AL \| 90 \| 84% \| \|  \| Quinine + AL \| 16 \| 15% \| \| **Admission outcome** \| Discharge \| 87 \| 81% \| \|  \| Abandonment \| 7 \| 7% \| \|  \| Fatality \| 11 \| 10% \| \|  \| Transfer to Quelimane \| 2 \| 2% \| \| **# hospitalization days** \| *Median (IQR)* \| *5 (3-16)* \| *--* \| |
