# Supplementary material for: The economic burden of malaria on households and the health system in a high transmission district of Mozambique
Source: Malar J. 2019 Nov 11;18:360. doi: 10.1186/s12936-019-2995-4 (PMC6849240; doi:10.1186/s12936-019-2995-4)
Supplement: Supplementary file 5 — Additional file 5. Differences in health system costs due to severe malaria according to sex, age and malaria season. [file 12936_2019_2995_MOESM5_ESM.docx]

| Additional file 5. Differences in health system costs due to severe malaria according to sex, age and malaria season. Mean (95% CI). US$ 2018 |
| --- |
| \|  \| Sex \| \| Age \| \| \| Malaria season \| \| \| \| --- \| --- \| --- \| --- \| --- \| --- \| --- \| --- \| --- \| \|  \| Male \| Female \| Under 5 \| \| Above 5 \| Rainy \| \| Dry \| \| Medical examinations \| 3.47  (3.30-3.63) \| 3.42  (3.26-3.58) \| 3.47  (3.29-3.66) \| \| 3.42  (3.27-3.56) \| **3.30**  **(3.20-3.40)** \| \| **3.72**  **(3.46-3.98)** \| \| Malaria diagnostic tests \| 8.73  (7.68-9.77) \| 8.27  (7.14-9.40) \| 8.49  (7.31-9.68) \| \| 8.52  (7.53-9.51) \| **7.56**  **(6.74-8.39)** \| \| **10.30**  **(8.90-11.70)** \| \| Malaria treatment \| 5.48  (2.12-8.85) \| 4.47  (3.43-5.52) \| **2.00**  **(1.47-2.54)** \| \| **7.83**  **(4.48-11.18)** \| 5.04  (2.36-7.73) \| \| 4.91  (3.52-6.31) \| \| Total costs \| 37.21  (28.05-46.38) \| 36.70  (24.39-49.00) \| 38.68  (24.98-52.37) \| \| 35.35  (28.34-42.37) \| **33.10**  **(24.32-41.88)** \| \| **44.28**  **(30.27-58.29)** \| \| MWM Test (total costs) \| z=0.76 \|  \| z=1.04 \|  \| \| z=2.82 \|  \| \| \| Test Power (bootstrapping) \| 44.60% \|  \| 29.50% \|  \| \| 99.95% \|  \| \| |
| *Note*: Mann-Whitney-Wilcoxon (MWW) non-parametric test for group differences. Bold numbers denote a statistically significant difference |
